# Augmented reality through head-mounted display for navigation of baseplate component placement in reverse total shoulder arthroplasty: a cadaveric study

**DOI:** 10.1007/s00402-021-04025-5

**Published:** 2021-07-02

**Authors:** Philipp Kriechling, Rafael Loucas, Marios Loucas, Fabio Casari, Philipp Fürnstahl, Karl Wieser

**Affiliations:** 1 Department of Orthopaedics, Balgrist University Hospital, Zurich, Switzerland; 2 Computer Assisted Research and Development Group, Balgrist University Hospital, Zurich, Switzerland

**Keywords:** Reverse total shoulder arthroplasty, Augmented reality, Head-mounted display, Navigation, Experimental, Cadaveric, Orthopedic surgery

## Abstract

**Background:**

To achieve an optimal clinical outcome in reverse total shoulder arthroplasty (RSA), accurate placement of the components is essential. The recently introduced navigation technology of augmented reality (AR) through head-mounted displays (HMD) offers a promising new approach to visualize the anatomy and navigate component positioning in various orthopedic surgeries. We hypothesized that AR through HMD is feasible, reliable, and accurate for guidewire placement in RSA baseplate positioning.

**Methods:**

Twelve human cadaver shoulders were scanned with computed tomography (CT) and RSA baseplate positioning was 3-D planned using dedicated software. The shoulders were prepared through a deltopectoral approach and an augmented reality hologram was superimposed using the HMD Microsoft HoloLense. The central guidewire was then navigated through the HMD to achieve the planned entry point and trajectory. Postoperatively, the shoulders were CT-scanned a second time and the deviation from the planning was calculated.

**Results:**

The mean deviation of the entry point was 3.5 mm  ± 1.7 mm (95% CI 2.4 mm; 4.6 mm). The mean deviation of the planned trajectory was 3.8°  ± 1.7° (95% CI 2.6°; 4.9°).

**Conclusion:**

Augmented reality seems feasible and reliable for baseplate guidewire positioning in reverse total shoulder arthroplasty. The achieved values were accurate.

**Supplementary Information:**

The online version contains supplementary material available at 10.1007/s00402-021-04025-5.

## Introduction

The use of reverse total shoulder arthroplasty (RSA) is spreading worldwide due to an aging population and an increasing variety of indications. Initially, RSA was indicated in patients suffering from rotator cuff arthropathy, but nowadays, RSA has become a valid option for massive rotator cuff tear, osteoarthritis, primary fracture treatment, or revision surgery [1–3]. To achieve optimal and reliable component placement, the implantation systems and techniques are being continuously improved. The optimal baseplate position is considered in a neutral version and neutral to slightly inferior inclination [4–6]. Especially in cases of severe glenoid deformation or complex revision surgeries, the accurate position of the baseplate can, however, be challenging due to bone deficiency and limited intraoperative visibility. Malpositioning of the components can potentially lead to complications like inferior scapular notching, loosening or instability, and unsatisfying clinical outcomes [7].

Computer-assisted pose tracking navigation or patient-specific instrumentation can be utilized to achieve the planned baseplate position. Both systems offer promising results but have a couple of disadvantages. Navigation systems are expensive to purchase and can be uncomfortable to use, potentially increasing surgical time. Patient-specific guides require a particular time of preoperative manufacturing and intraoperative specific handling [8, 9]. Recently, the technology of augmented reality (AR) through a head-mounted display (HMD) has been introduced [10]. This technology seems easy to use and comparably cheap without the disadvantage of long preoperative preparation time. The current literature on this topic is very sparse, especially regarding shoulder surgery. Berhouet et al. [11] described the 3D projection of a hologram of a reconstructed scapula. Gregory et al. [12] presented the intraoperative projection in one case. Both did not utilize the HMD for navigation but could prove the high potential of AR in RSA surgery. In a previous study, we analyzed the feasibility of HMD in ten 3D-printed bone models and found a mean deviation from the entry point of 2.26 mm ± 1.11 mm. The deviation from the planned vector was 2.74° ± 1.25° [13].

This study aimed to investigate the feasibility of AR navigation through HMD to guide the RSA baseplate positioning in a cadaveric study. We hypothesized that this new technology could be reliably used, providing high accuracy of implant position in a cadaveric setting.

## Methods

### Ethical statement

The study has been performed in accordance with the ethical standards in the 1964 Declaration of Helsinki. The study was accepted by the cantonal ethics committee of Zurich under the number 2017-00874. All experiments were conducted in a human cadaveric laboratory, access was granted only to people directly involved in the experiments.

### Study design

The study was conducted with one group of twelve fresh-frozen human cadaveric scapulae with the adjacent humerus. According to the planning, one orthopedic surgeon (P.K.) placed one guidewire for baseplate positioning in each glenoid under AR navigation. The planning and analysis were done using computed tomography.

### Experimental procedures

The experimental procedures were all conducted in a standardized setting at the same time of the day on six consecutive days. All twelve deep-frozen cadavers were scanned using computed tomography (Siemens Somotom Edge Plus, Germany) in 0.5 mm slice increments. Each scapular was segmented separately with global thresholding and the region growing tool in a standard segmentation software (MIMICS 23, Leuven, Belgium). The 3D models were exported as *.stl-Files and imported into our institution’s standard 3D-Planning software (CASPA, Balgrist CARD, Zurich, Switzerland). The guidewire position was planned in the computer software to reach inferior baseplate position in neutral version and inclination. The data were then prepared and converted using Unity Software (Unity Technologies, San Francisco, CA, USA, Version, 2019.1.7) and Microsoft Visual Studio (version Community 2017, Microsoft Corporation, Redmond, WA, USA) and then set up on the HoloLens.

Each scapula was placed in a self-manufactured holding device at the institution’s cadaver laboratory. The human cadaveric models contained the full scapula and humeral bone with all adjacent muscles, soft tissue, and skin to simulate natural conditions. For all individuals, a deltopectoral approach was utilized. The subscapularis muscle was sharply dissected at the lesser tuberosity and held out of the operative field with retaining cords. The supraspinatus muscle was slightly dissected at the footprint and kept dorsally. Then the joint capsule was resected. Subsequently, the humerus could be dorsally sub-luxated and held away using retractors. The glenoid came into full view.

Then the navigation was started. A 1.6 mm Kirschner-wire was placed with a drilling machine (PSR14,4 LI-2, Bosch AG, Gerlingen, Germany) in every glenoid using Microsoft HoloLense for augmented reality navigation through a head-mounted display (described below).

The achieved Kirschner-wire position was analyzed using a second computed tomography. The scanned scapulae were segmented as described above and imported into our institution’s planning software (CASPA, Balgrist CARD, Zurich, Switzerland) to overlay the preoperative and postoperative scapula by nearest iterative point cloud analysis [14]. The deviation from the planning (entry point in mm and trajectory in a three-dimensional angle error) was calculated.

### Surgical navigation

The Microsoft Head-Mounted Display HoloLense Version 1 (Microsoft, Redmond, WA, USA) was employed in the study to register the position of the scapula in the room, to set up a hologram exactly over the operative site to navigate the guidewire. Voice commands and gestures controlled the HoloLense.

**Fig. 1:**
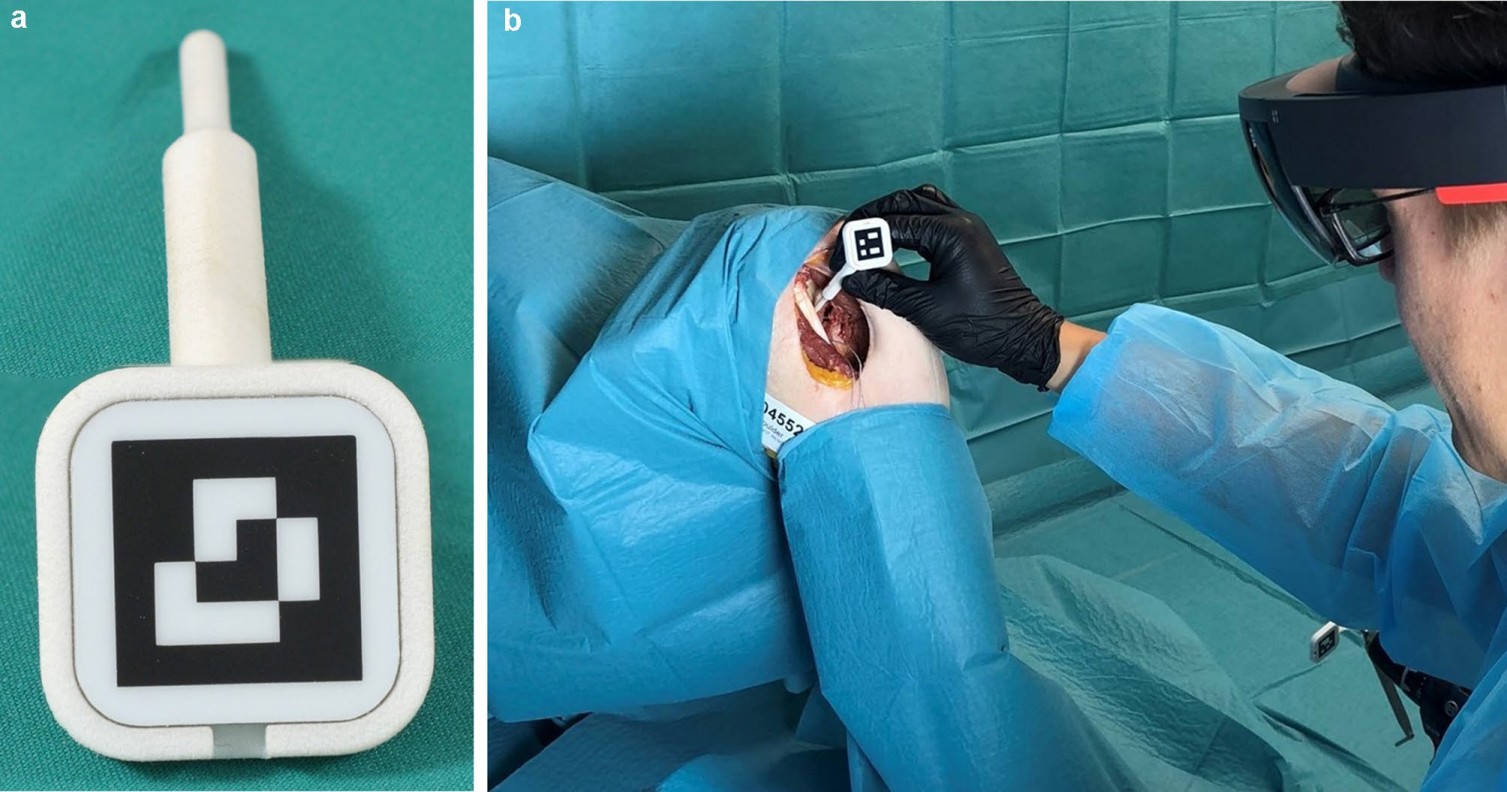
The registration process necessary to achieve hologram overlay. **a** The fiducial marker to detect and track the surface. **b** The intraoperative application

**Fig. 2:**
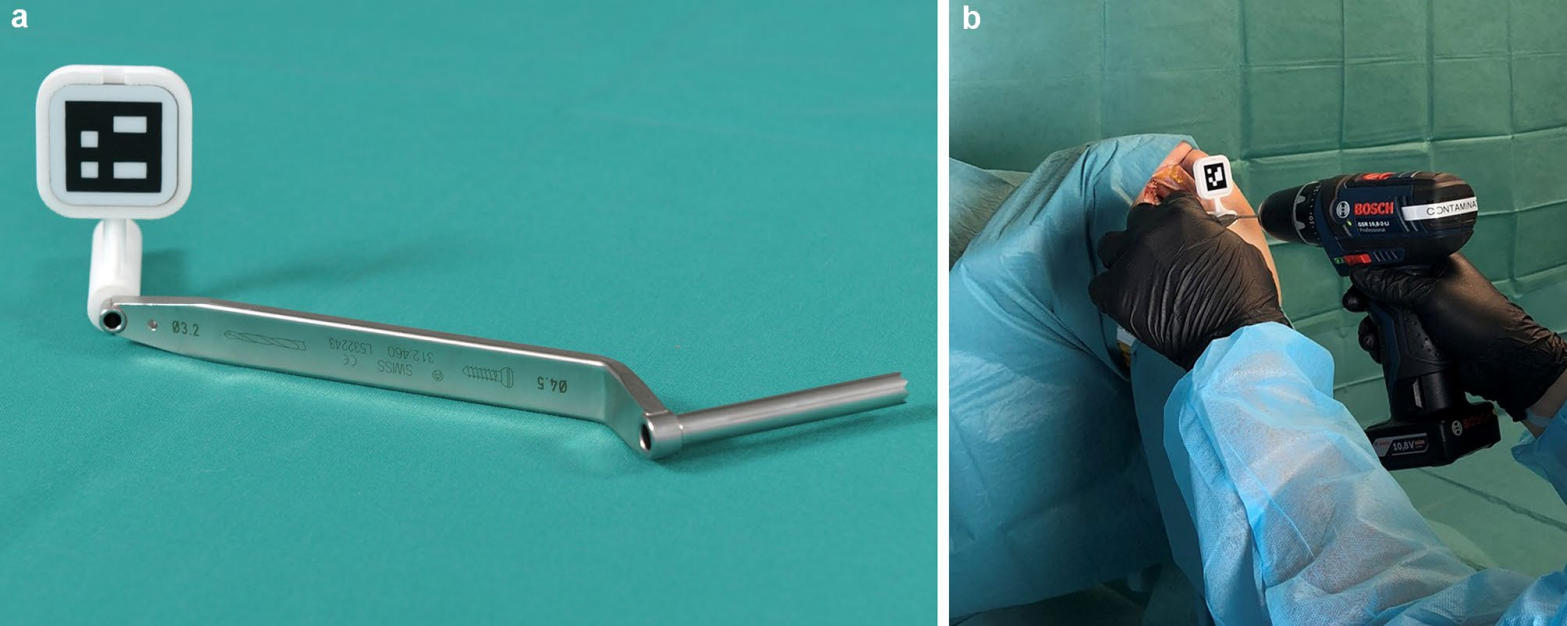
The navigation process with the **a** drill guide for head-mounted display navigation. **b** The intraoperative application

**Fig. 3:**
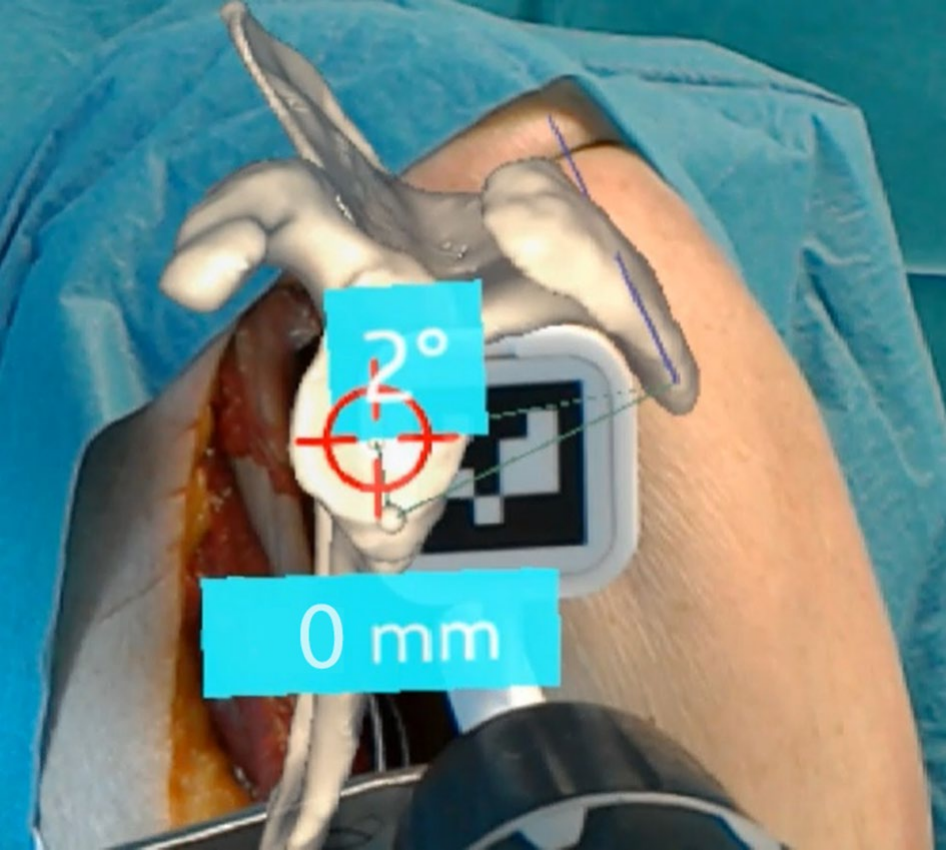
The surgeon’s view and is obtained from the head-mounted display camera. The superimposed hologram shows the scapula with the planned entry point and the deviation in millimeters. The planned trajectory of the guidewire and the current deviation is displayed in degrees

**Fig. 4:**
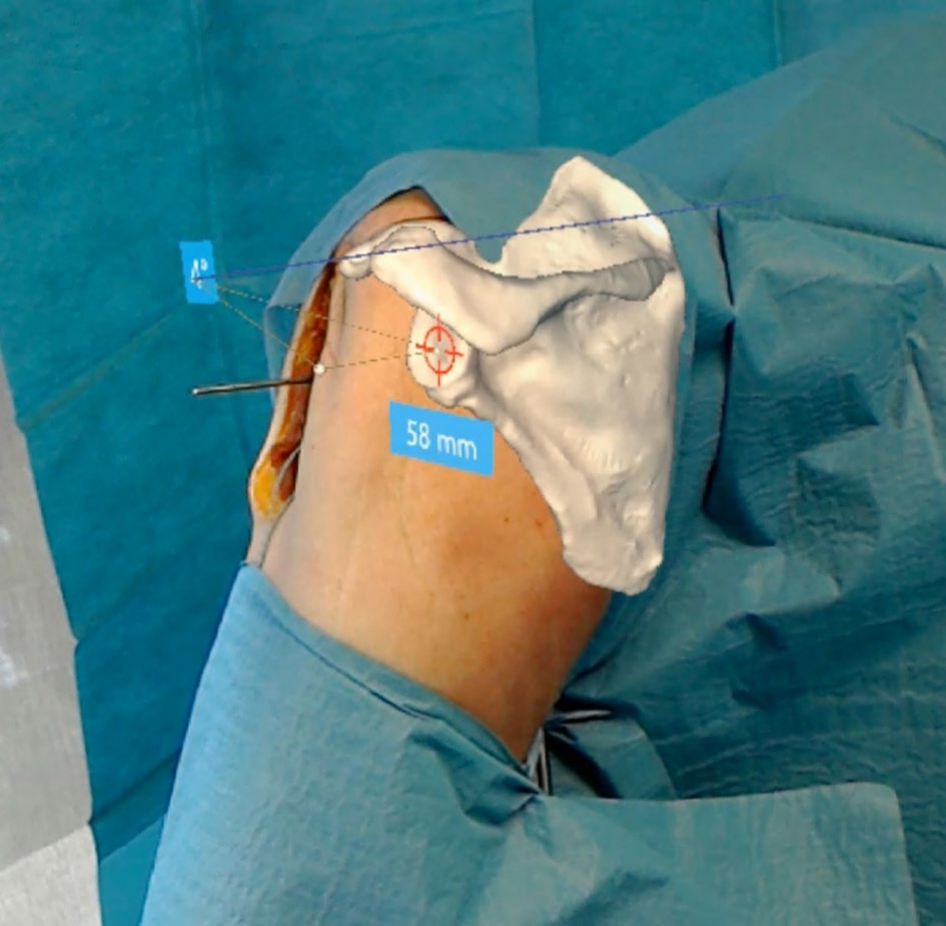
The surgeon’s navigation perspective from another angle. The scapula is permanently superimposed on the real anatomy after registration. This allows viewing from several angles

The scapula position was registered using a custom-made fiducial marker and stereo-tracking, as described before [15] (Fig. 1). First, the rough orientation was defined through the location of the acromion, coracoid, and glenoid. Those points were defined in the preoperative planning using our institution’s software (CASPA). Second, the fine adjustment was achieved by tracing the glenoids’ surface. The exact location of the hologram was then calculated and superimposed as augmented reality to the operative site. After confirming the optimal position, the navigation process was started (Fig. 2, Video 1). A virtual model of the scapula appeared in the surgeon’s field of vision. The drill sleeve with a fiducial marker was used to display the planned entry point with deviation in millimeters and the planned vector with deviation in degree (Fig. 3). With this information, the target wire could be placed (Fig. 4).

### Outcome parameters

Outcome measures were deviation from the planned guidewire direction (vector), deviation of the glenoid side entry point, and the number of attempts to get optimal hologram overlay.

The 3D angular error (AE) was calculated using the direction vector of the planned ($$\overset{\lower0.5em\hbox{$\smash{\scriptscriptstyle\rightharpoonup}$}} {A}$$) and executed ($$\overset{\lower0.5em\hbox{$\smash{\scriptscriptstyle\rightharpoonup}$}} {B}$$) trajectories by applying the following formula:

$${\text{AE}} = \cos ^{{ - 1}} \left( {\frac{{\overset{\lower0.5em\hbox{$\smash{\scriptscriptstyle\rightharpoonup}$}} {A} ~ \cdot ~\overset{\lower0.5em\hbox{$\smash{\scriptscriptstyle\rightharpoonup}$}} {B} }}{{\left\| {\overset{\lower0.5em\hbox{$\smash{\scriptscriptstyle\rightharpoonup}$}} {A} } \right\|~~\left\| {\overset{\lower0.5em\hbox{$\smash{\scriptscriptstyle\rightharpoonup}$}} {B} } \right\|~}}} \right)$$

The entry point error (TE) was calculated as Euclidean distance between the centers of the planned (*x*_1_, *y*_1_, *z*_1_) and achieved (*x*_2_, *y*_2_, *z*_2_) entry points using the following formula:

$${\text{TE}} = ~\sqrt {\left( {x_{1} - x_{2} } \right)^{2} + ~\left( {y_{1} - y_{2} } \right)^{2} + ~\left( {z_{1} - z_{2} } \right)^{2} ~}$$

**Fig. 5:**
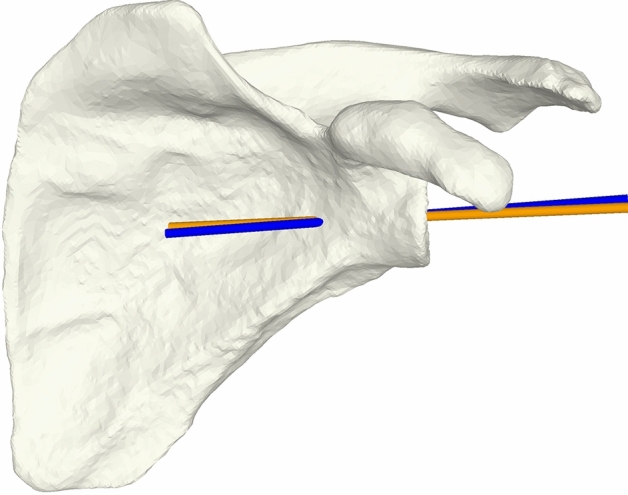
Postoperative calculation of achieved deviation. Preoperative planning is represented by the orange vector, which was planned after the first CT imaging. The blue vector corresponds to the intraoperatively placed wire, determined by second CT imaging

The analysis is shown in Fig. 5.

Adverse events were recorded. They were defined as technical or patient-specific events. Technical issues included problems recognizing the scapula or the tracker by the Holo Lense or the impossibility of navigating the guidewire. Patient-specific complications included intraoperative fractures during drilling or damage to critical structures such as nerves or vessels.

### Statistical analysis

For statistical calculations, SPSS v23.0 (IBM, New York, United States of America) was utilized. Descriptive statistics are given as mean ± standard deviation (95% confidence interval). Comparison of nonparametric data was performed using the Mann–Whitney *U* test.

## Results

We included all twelve shoulders in our analysis. The mean age was 73 ± 2 (71–75) years, eight of twelve shoulders were male with four right and eight left shoulders.

All twelve guidewires were placed without any technical problems. The software worked without problems. Only one registration attempt to overlay the image correctly was necessary for eleven of twelve specimens, and two attempts were required for the other specimen. The mean deviation from the planned entry point was 3.5 mm ± 1.7 mm (95% CI 2.4 mm; 4.6 mm). The mean deviation from the planned trajectory was 3.8° ± 1.7° (95% CI 2.6°; 4.9°) (Table 1).

**Table 1:** Results of the baseplate navigation accuracy using augmented reality through head-mounted display

ID	3D vector	Entry point	Attempts	Side
1	1.0°	3.3 mm	1	Left
2	3.2°	5.7 mm	1	Left
3	7.8°	2.3 mm	2	Left
4	3.2°	4.3 mm	1	Right
5	3.4°	4.0 mm	1	Right
6	4.7°	0.6 mm	1	Left
7	4.3°	2.3 mm	1	Left
8	4.9°	5.4 mm	1	Left
9	4.7°	1.9 mm	1	Left
10	3.4°	4.0 mm	1	Right
11	1.0°	6.2 mm	1	Left
12	3.3°	1.6 mm	1	Right
Mean 95% CI	3.8° ± 1.7° (95% CI 2.6°; 4.9°)	3.5 ± 1.7 mm (95% CI 2.4; 4.6)	1 ± 0.3	

The values 3D vector and entry point describe the deviation from the planning measured by comparing the preoperatively planned vector with the postoperatively achieved vector using computed tomography. Attempts reflect the number of registration processes to achieve adequate hologram overlay

*CI* confidence interval, *mm* millimeter

The side (8 left shoulders, 4 right shoulders) did not influence the deviation from the planned vector (*p* < 0.46) and planned entry point (*p* = 1.0) in the setting of a right-handed surgeon. There was no subjective difference in treatment of right or left shoulders for the surgeon using HMD-assisted navigation. No adverse event occurred.

## Discussion

The conducted study confirmed our hypotheses that (1) the use of AR navigation to position the glenoid baseplate component in RSA is feasible and (2) can achieve good accuracy in a cadaveric setting. The mean deviation from the planned entry point was 3.5 mm and the deviation from the planned 3D trajectory was 3.8°. We saw high reliability with mostly one registration attempt necessary to achieve correct hologram superimposition.

To the best of our knowledge, this is the first report in the current literature that describes glenoid guidewire positioning utilizing AR with an HMD as a navigation device in a cadaveric model. We previously analyzed the use of AR in ten 3D-printed scapulae and found a mean deviation from the entry point of 2.26 mm ± 1.11 mm. The deviation from the planned vector was 2.74° ± 1.25° [13]. Berhouet et al. described the application of augmented reality to display a three-dimensional reconstruction of the glenoid and the adjacent scapula using a head-mounted display [11]. On a technical note, Gregory et al. described using a head-mounted display intraoperatively in one single patient. They could drag and drop the scapula in the operative field, display the computed tomography data, and conference with other surgeons remotely [12].

Our study results should be compared to other AR applications. Wang et al. [16] described AR through HMD for navigation of pedicle screws with a deviation trajectory of 2.9° ± 1.1° and a deviation from the entry point of 2.7 ± 1.2 mm in 6 human cadavers. Müller et al. [17] tested the AR navigation through a head-mounted display for pedicle screw instrumentation in human cadavers. The results were comparable to a state-of-the-art pose-tracking system. Molino et al. [18] described in another human cadaver study non-inferiority of AR using a HMD in comparison to freehand, manual computer-navigated, and robotics-assisted computer-navigated pedicle screw placement. Other studies in the current literature reported mainly using HMD to show information in the clinical or cadaveric setting, less on the function of AR navigation through HMD. When used as a replacement for a free-standing monitor, the duration of surgery could be shortened with a lower radiation dose and increased concentration on the surgical area [19, 20].

The optimal application area for HMD-navigated component placement has yet to be defined. Currently, especially for severe glenoid deformities, PSI or computer-supported pose tracking systems are well-validated systems. A recently published meta-analysis of 277 shoulders showed a 2D accuracy of 2.7° ± 0.5° for the version and 1.9° ± 0.4° for the inclination with a deviation of the entry point of 1.1 mm ± 0.2 mm when using PSI. This was superior to the standard freehand method (version 5.9° ± 1.1°, inclination 5.8° ± 1°, entry point 2 mm ± 0.4 mm) [8]. Another recently published meta-analysis from 2019 including seven studies revealed a mean deviation from the planning of 3.2° (95% CI − 4.6° to + 4.6°) and 1.2° (95% CI − 4.6° to + 1.3°) for version and inclination, respectively. The offset deviation was 0.2 mm (95% CI − 4.6 to 0.4 mm). Since they included only studies comparing PSI to freehand, no statistically significant difference was detected, albeit the deviation for freehand baseplate positioning was larger [21]. The disadvantages of PSI are the costs and long preparation time required for the individual planning and production of the guides. AR through HMD offers a straightforward application in the operating room with low costs for the setup.

Computer navigation using pose-tracking reaches similar results but can be complex and error-prone in the setup. Stübig et al. compared 15 navigated and 12 conventional glenoid baseplate implantations and detected no difference for inclination, but a significantly better version with 1.6° ± 4.5° and 11.5° ± 6.5° for the navigated and conventional group, respectively [22]. Wang described 25 prospectively followed cases with a mean deviation from the planing for version of 3° ± 2° and for inclination of 5° ± 3° [23].

The presented results of PSI and pose-tracking navigation are comparable to our study results. A further development of navigation using AR through HMD should be promoted, knowing better hardware will be implemented.

Several limitations have to be discussed. (1) It was a human cadaver study without in vivo problems like bleeding, the setting of an operation room, or the movement of the chest and scapula during breathing. (2) The time necessary to navigate the guidewires was not measured. This fact was accepted knowingly, as the study aimed to analyze the feasibility and accuracy in a cadaveric setting. In the study planning, time recording was rejected to avoid influence on results by overhasty insertion of the wires. Nevertheless, a surprisingly low time expenditure can be reported as a maximum of two registration attempts were necessary to overlay the hologram in each individual. (3) We decided to conduct the study without a control group to test the proposed navigation method against the optimal position, calculating the deviation from the planned entry point and planned trajectory. Future studies are mandatory to prove the superiority of this navigation system over freehand guidewire positioning, ideally performed by multiple surgeons. In the further course, an application should be implemented in clinical studies and a functional extension for additional placement of the glenoid screws at the site of maximum bone strength.

The results of this study have to be interpreted accepting the original purpose of Microsoft’s Holo Lense as multimedia and entertaining device. Primarily, it was not invented to function as a medical device with a high necessity of accuracy and reliability. Nevertheless, a valuable and reliable application could be shown in this study. Upcoming AR devices will probably be designed to serve medical purposes and can thus be equipped with high-end and eventually more expensive hardware.

## Conclusion

Baseplate navigation in reverse total shoulder arthroplasty using augmented reality through a head-mounted display seems feasible, showing high accuracy in a cadaveric shoulder model.

## Supplementary Information

Below is the link to the electronic supplementary material.

Supplementary file1 Video 1: Illustrates the utilization of the glenoid guide wire navigation using augmented reality through a head-mounted display. First, the rough orientation has to be defined. Then the exact expansion of the glenoid has to be defined. The navigation mode displays the planned entry point and the planned vector. (MP4 42083 kb)
